# Characterization of Phenolic Constituents from Ephedra Herb Extract

**DOI:** 10.3390/molecules18055326

**Published:** 2013-05-10

**Authors:** Yoshiaki Amakura, Morio Yoshimura, Saori Yamakami, Takashi Yoshida, Daigo Wakana, Masashi Hyuga, Sumiko Hyuga, Toshihiko Hanawa, Yukihiro Goda

**Affiliations:** 1 Department of Pharmacognosy, College of Pharmaceutical Sciences, Matsuyama University, 4-2 Bunkyo-cho, Matsuyama, Ehime 790-8578, Japan; E-Mails: myoshimu@cc.matsuyama-u.ac.jp (M.Y.); s_yamakami@cc.matsuyama-u.ac.jp (S.Y.); tyoshida@gem.e-catv.ne.jp (T.Y.); 2 Division of Pharmacognosy, Phytochemistry and Narcotics, National Institute of Health Sciences, 1-18-1 Kamiyoga, Setagaya-ku, Tokyo 158-8501, Japan; E-Mails: d-wakana@nihs.go.jp (D.W.); goda@nihs.go.jp (Y.G.); 3 Division of Biological Chemistry and Biologicals, National Institute of Health Sciences, 1-18-1 Kamiyoga, Setagaya-ku, Tokyo 158-8501, Japan; E-Mail: mhyuga@nihs.go.jp; 4 Department of Clinical Research, Oriental Medicine Research Center of Kitasato University, 5-9-1 Shirokane, Minato-ku, Tokyo 108-8642, Japan; E-Mails: hyuga-s@insti.kitasato-u.ac.jp (S.H.); hanawa-t@insti.kitasato-u.ac.jp (T.H.)

**Keywords:** Ephedra herb, herbacetin 7-*O*-neohesperidoside, phenolics, Mao, Kampo

## Abstract

Nine known compounds: *trans*-cinnamic acid, catechin, syringin, epicatechin, symplocoside, kaempferol 3-*O*-rhamnoside 7-*O*-glucoside, isovitexin 2-*O*-rhamnoside, herbacetin 7-*O*-glucoside, and pollenitin B and a new flavonoid glycoside, characterized as herbacetin 7-*O*-neohesperidoside (**1**) on the basis of spectroscopic analysis and chemical evidence, were isolated from a traditional crude drug, “Ephedra herb extract”. Compound **1** had no effects on HGF-induced motility, whereas herbacetin, which is an aglycone of **1**, significantly inhibited it.

## 1. Introduction

Ephedra herb (Japanese name: “Mao”), is defined in the Japanese Pharmacopoeia (JP) as the terrestrial stem of *Ephedra sinica* Staf, *E. intermedia* Schrenk et C.A. Meyer or *E. equisetina* Bunge (Ephedraceae), [1]. It is one of the most important crude drugs of Kampo prescriptions such as Maoto, Kakkonto, Makyokansekito, and Syoseiryuto, which have been used as anti-tussive, expectorant, anti-pyretic analgesic, and bronchodilator agents [2]. It is well known that the traditional biological properties of Ephedra herb are attributable in a large part to ephedrine-type alkaloids such as ephedrine, pseudoephedrine, and norephedrine [3].

Recently, our research group reported that the extract product of Ephedra herb (“Ephedra herb extract”) inhibits the hepatocyte growth factor (HGF)-induced motility of human breast cancer MDA-MB-231 cells through the suppression of c-Met tyrosine phosphorylation [4]. In that paper, we discussed that the active components in the extract might be non-alkaloid substances because ephedrine had no effect on HGF-induced motility. Reports on the constituents in the extract besides ephedrine-type alkaloids are limited, although condensed tannins [5] and flavonoids were found in some *Ephedra* species [6,7]. In order to explore the active components associated with the antitumor expression shown by Ephedra herb extract, we have investigated the phenolic compounds in the extract, and characterized 10 compounds, including a new flavonoid, which is the subject of this paper.

## 2. Results and Discussion

**Figure 1:**
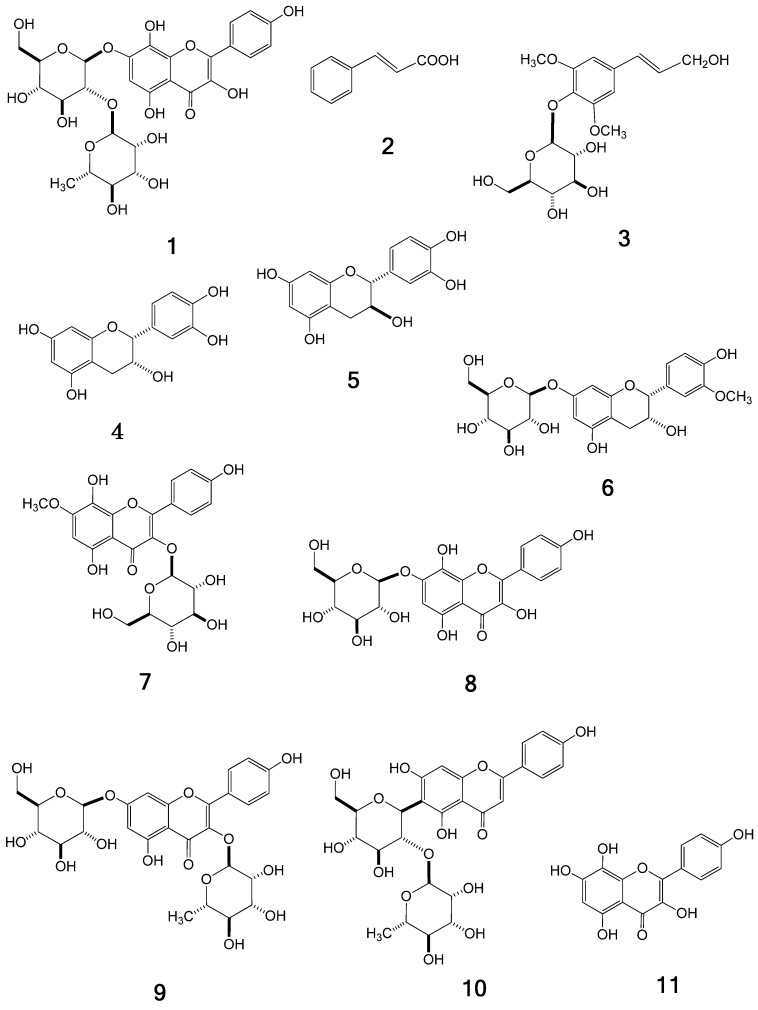
Structures of compounds **1**–**11**.

The Ephedra herb extract, purchased from Tsumura & Co., was dissolved in water and partitioned with *n*-hexane, ethyl acetate (EtOAc), and *n*-butanol (BuOH) to give the respective *n*-hexane, EtOAc, BuOH, and water extracts. The EtOAc and *n*-BuOH extracts, which showed the presence of polyphenolics in HPLC, were chromatographed using Diaion HP-20, Sephadex LH-20, Toyopearl HW-40, MCI-gel CHP-20P, and/or YMC GEL ODS-AQ with aqueous methanol (MeOH) in a stepwise gradient mode. The fractions showing similar HPLC patterns were combined and further purified by column chromatography with aqueous MeOH, to afford a new compound **1**, together with the nine known compounds *trans*-cinnamic acid (**2**) [8], syringin (**3**) [9], catechin (**4**) [10], epicatechin (**5**) [10], symplocoside (**6**) [11], pollenitin B (**7**) [12], herbacetin 7-*O*-glucoside (**8**) [6], kaempferol 3-*O*-rhamnoside 7-*O*-glucoside (**9**) [13], and isovitexin 2-*O*-rhamnoside (**10**) [14] (Figure 1), which were identified by direct comparison with authentic specimens or by spectral comparisons with data reported in the literature.

**Figure 2:**
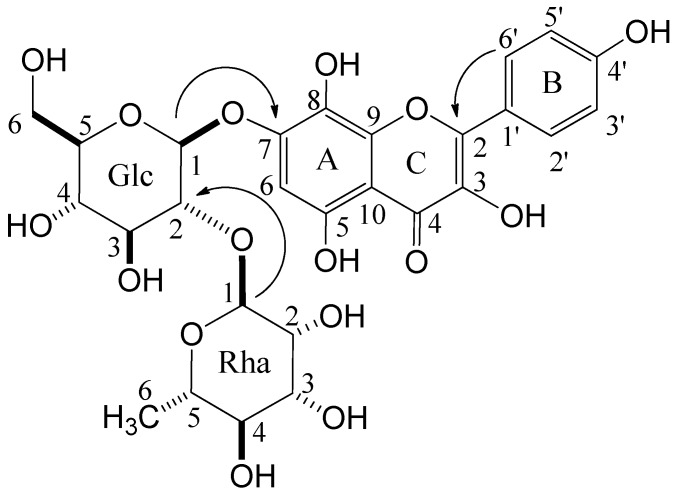
Key HMBC correlations of compound **1**.

Compound **1** was isolated as a light brown amorphous powder. Its molecular formula was assigned as C_27_H_3__0_O_1__6_ from its HR-ESI-MS (*m/z* 609.1466 [M−H]^−^; calcd. for C_27_H_3__0_O_1__6_-H: 609.1461) and ^13^C-NMR (27 ^13^C signals) spectrum. The UV spectrum (MeOH) showed maxima at 206, 223sh, 274, 332, and 382, which are characteristic of flavonoids [12]. The presence of a herbacetin skeleton in **1** was indicated by A_2_B_2_-type doublets at δ 6.90 and 8.20 (2H, each d, *J* = 9.0 Hz) and a singlet at δ 6.62 (1H, s) in the ^1^H-NMR spectrum, and 15 *sp*^2^-carbon resonances assignable to B-ring [δ 116.3 (2C), 123.8, 131.1 (2C), 160.8] and A/C-rings (δ 99.5, 106.4, 128.9, 137.4, 146.3, 148.8, 151.9, 153.8, 177.8) in the ^13^C-NMR spectrum (Table 1). In the aliphatic proton region, a methyl proton signal [δ 1.90 (d, *J* = 6 Hz)] and two doublets [δ 5.20 (d, *J* = 7.5 Hz) and 5.29 (d, *J* = 1.5 Hz)] attributable to anomeric protons of sugar units were observed, together with 10 mostly overlapped proton signals at δ 3.3–3.9, suggesting the presence of two sugar residues. The chemical shifts of the *sp*^3^-carbon signals in the ^13^C-NMR spectrum were consistent with those of rhamnose and glucose in compound **9** or other analogs [13]. Compound **1** was thus presumed to be herbacetin rhamnoglucoside, which was verified by production of herbacetin (**11**) [15] as the aglycone upon acid hydrolysis. The sugar components liberated in the hydrolysis were identified as D-glucose and L-rhamnose according to the previously reported method [16]. Glycosidic linkages in **1** were determined as β- and α-configuration for D-glucose and L-rhamnose, respectively, based on the coupling constants of each anomeric proton signal. The linking position of each unit was confirmed by cross-peaks between glucose H-1 (δ 5.20) and C-7 (δ 151.9) of herbacetin, and rhamnose H-1 (δ 5.29) and C-2 (δ 80.5) of glucose in HMBC (Figure 2). Therefore, **1** was established as herbacetin 7-*O*-neohesperidoside.

**Table 1:** ^1^H- (500 MHz) and ^13^C-NMR (126 MHz) data of compound **1** measured in MeOH-*d*_4_.

Position	δ_C_	δ_H_ ( *J* in Hz)
2	148.8	
3	137.4	
4	177.8	
5	153.8	
6	99.5	6.62 (s)
7	151.9	
8	128.9	
9	146.3	
10	106.4	
1'	123.8	
2'	131.1	8.20 (d, *J* = 9)
3'	116.3	6.90 (d, *J* = 9)
4'	160.8	
5'	116.3	6.90 (d, *J* = 9)
6'	131.1	8.20 (d, *J* = 9)
glucose (Glc)-1	101.3	5.20 (d, *J* = 7.5)
2	80.5	3.75 (dd, *J* = 7.5, 10)
3	78.6 ^a^	3.34–3.92 ^c^
4	72.2 ^b^	3.34–3.92 ^c^
5	78.2 ^a^	3.34–3.92 ^c^
6	62.1	3.34–3.92 ^c^
rhamnose (Rha)-1	102.9	5.29 (d, *J* = 1.5)
2	71.1	3.98 (d, *J* = 1.5, 3.5)
3	72.3 ^b^	3.34–3.92 ^c^
4	70.5	3.34–3.92 ^c^
5	74.0	3.34–3.92 ^c^
6	17.8	1.90 (3H, d, *J* = 6)

^a,b^ Assignments may be interchanged; ^c^ Overlapped signals.

**Figure 3:**
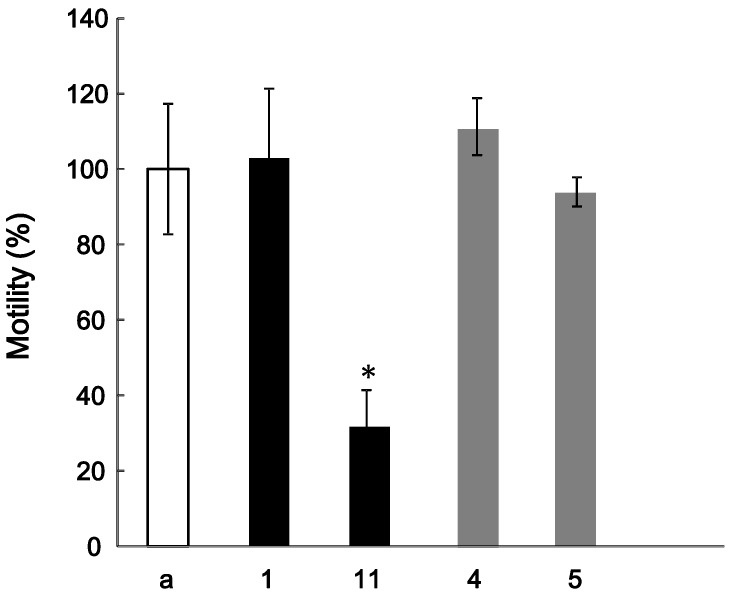
Effect of compounds **1**, **4**, **5**, and **11** on the HGF-induced motility of MDA-MB-231 cells. The cells were suspended in DMEM (a) alone or with **1**, **11**, **4**, or **5** (**1** and**11**: 4 μg/mL, **4** and **5**: 10 μg/mL), and poured into the upper well of the Transwell. The lower well contained 600 µL of DMEM containing 50 ng/mL HGF. At 20 h, the cells migrating into the lower well were counted. * p < 0.05 *vs*. the control.

The effect of **1** on the HGF-induced motility of MDA-MB-231 cells was estimated with a method similar to that in the previous report [4]. However, the tested compound had no effects on HGF-induced motility. In general, it is reported that glycosides in Kampo medicines are metabolized to aglycones by intestinal flora [17]. We thus assessed the effects of **11**, which is an aglycone of **1**, together with **4** and **5** on the HGF-induced motility. The HGF-induced motility was significantly reduced by the addition 4 μg/mL of **11**; however, **4** and **5** had no effects on the HGF-induced motility (Figure 3). Thus, it is suggested that **11** is one of the active components associated with the antitumor effect shown by the Ephedra herb extract. Further investigation of other compounds, including other fractions in the Ephedra herb extract, is currently in progress.

## 3. Experimental

### 3.1. General

Optical rotations were measured with a JASCO P-1020 digital polarimeter (JASCO, Tokyo, Japan). UV spectra were recorded on a Shimadzu UVmini-1240 (Shimadzu, Kyoto, Japan). Electrospray ionization (ESI)-MS, and high-resolution (HR) ESI-MS spectra were obtained using a micrOTOF-Q (Bruker Daltonics, MA, USA) mass spectrometer with acetonitrile as the solvent. ^1^H- and ^13^C-NMR spectra were recorded on a Brucker AVANCE500 instrument (Bruker BioSpin, Billerica, MA, USA) (500 MHz for ^1^H and 126 MHz for ^13^C) or JEOL ECA-800 instrument (JEOL, Tokyo, Japan) (800 MHz for ^1^H and 201 MHz for ^13^C), and chemical shifts are given in ppm values relative to those of the solvents [MeOH-*d*_4_ (δ_H_ 3.30; δ_C_ 49.0)] on a tetramethylsilane scale. The standard pulse sequences programmed for the instrument (AVANCE 500) were used for each 2D measurement (COSY, HSQC, and HMBC).* J*_CH_ was set at 10 Hz in HMBC. Column chromatography was carried out with Diaion HP-20, MCI-gel CHP-20P (Mitsubishi Chemical Co., Tokyo, Japan), YMC gel ODS (YMC Co., Ltd., Kyoto, Japan), and Sephadex LH-20 (GE Healthcare, Little Chalfont, England), respectively. The Sep-Pak Plus cartridge was from Waters (MA, USA). Normal-phase (NP) HPLC was conducted on a YMC-Pack SIL A-003 (YMC Co., Ltd.) column (4.6 i.d. × 150 mm) developed with *n*-hexane-MeOH-tetrahydrofuran-formic acid (55:33:11:1) containing oxalic acid (450 mg/L) (flow rate: 1.5 mL/min; 280 nm UV detection). Reversed-phase (RP) HPLC conditions were as follows: (Condition 1) column, l-column ODS (5 μm, 150 × 2.1 mm i.d.) (Chemicals Evaluation and Research Institute, Tokyo, Japan); mobile phase, solvent A was 5% acetic acid and solvent B was acetonitrile (0–30 min, 0–50% B in A; 30–35 min, 50–85% B in A; 35–40 min, 85–85% B in A); injection volume, 2 μL; column temperature, 40 °C; flow-rate, 0.3 mL/min; detection, 280 nm. 

### 3.2. Samples and Reagents

Ephedra herb (from the aerial parts of *E. sinica*) extract was obtained from Tsumura & Co. (Tokyo, Japan). L-Cysteine methyl ester hydrochloride and *o*-tolyl isothiocyanate were purchased from Wako Pure Chemical Industries (Osaka, Japan). All other reagents were of analytical grade.

### 3.3. Extraction and Isolation

Ephedra herb extract (300 g) were dissolved in water (5 L) and extracted with *n*-hexane (4 L), EtOAc (12 L), and *n*-BuOH (12 L) successively to give *n*-hexane (92 mg), EtOAc (13.2 g), *n*-BuOH (65.7 g), and water (161.1 g) extracts. The EtOAc extract (0.5 g) was chromatographed over MCI-GEL CHP-20P with MeOH/H_2_O (0:100 → 10:90 → 20:80 → 30:70 → 40:60 → 100:0) in a stepwise gradient mode. The fractions showing similar HPLC patterns were combined and further purified by column chromatography over Sephadex LH-20 with EtOH and YMC GEL ODS-AQ with aqueous MeOH to afford catechin (**4**) (45 mg) and *trans*-cinnamic acid (**2**, 3 mg). The *n*-BuOH extract (60 g) was separated by column chromatography over Diaion HP-20 with aqueous MeOH 20–60% MeOH eluates (8.6 g) and further subjected to column chromatography over Sephadex LH-20, MCI-GEL CHP-20P, and/or YMC GEL ODS-AQ with aqueous MeOH to yield syringin (**3**, 26 mg), catechin (**4**, (9.3 mg), epicatechin (**5**, 10.8 mg), symplocoside (**6**, 9.7 mg), pollentitin B (**7**, 8.6 mg), herbacetin 7-*O*-glucoside (**8**, 3.4 mg), kaempferol 3-*O*-rhamnoside 7-*O*-glucoside (**9**, 4 mg), isovitexin 2-*O*-rhamnoside (**10**, 133.2 mg), and herbacetin 7-*O*-neohesperidoside (**1**, 6.6 mg). These compounds were identified by direct comparison with authentic specimens or by comparison of their spectral data with those reported in the literature. The physical data of new compound **1** are as follows:

*Herbacetin 7-O-neohesperidoside* (**1**): A light brown amorphous powder. UV λ_max_ (MeOH) nm (logε): 206 (4.42), 223sh (4.29), 274 (4.30), 332 (4.09), 382 (4.04). [α]^23^_D_ −96.5° (*c* 1.0, MeOH). ^1^H-NMR (500 MHz, MeOH-*d*_4_) and ^13^C-NMR (126 MHz, MeOH-*d*_4_) data provided in Table 1. HR-ESI-MS *m/z*: 609.1466 ([M−H]^−^, Calcd. for C_27_H_3__0_O_16_-H: 609.1461). 

### 3.4. Determination of Sugar Configuration

The sugar configuration was determined using previously described methods [16]. Compound **1** (1.0 mg) was hydrolyzed by heating in 0.5 M HCl (0.2 mL) and neutralized with Amberlite IRA400. After evaporation and drying, the residue was dissolved in pyridine (0.2 mL) containing L-cysteine methyl ester hydrochloride (1.0 mg) and heated at 60 °C for 1 h. After cooling, *o*-tolyl isothyocyanate (1.0 mg) in pyridine (0.2 mL) was added to the mixture and heated again at 60 °C for 1 h. The reaction mixture was directly analyzed by RP-HPLC. The peaks coincided with those of derivatives similarly prepared from D-glucose and L-rhamnose.

### 3.5. Acid Hydrolysis of ***1***

A solution of **1** (1.5 mg) in 0.1 M HCl (0.5 mL) was heated in a boiling water bath for 5 h. After cooling, the reaction mixture was adsorbed on the Sep-Pak tC18 Plus cartridge (900 mg). After washing with water, the product was eluted with MeOH, and the concentrated solution was analyzed by reversed-phase HPLC to detect herbacetin (**11**, *R_t_* 27.3 min), which was identified by co-chromatography with an authentic sample [15].

### 3.6. Transwell Migration Assay

The MDA-MB-231 cells, obtained from the American Type Culture Collection (Manassas, VA, USA), were suspended at 5 × 10^4^ cells in 100 μL of DMEM containing 4 or 10 μg/mL of the tested compounds, and poured into the upper well of the Transwell permeable support system (Corning Incorporated, Acton, MA, USA). Six hundred microliters of DMEM medium containing 50 ng/mL of HGF (R&D Systems, Minneapolis, MN, USA) was added to the lower well. The transwell was then incubated for 20 h at 37 °C, and the number of cells that had migrated to the lower well was counted. Motility (%) is {(the number of migrated cells with the tested compound/the number of migrated cells without it) × 100}. Each assay was performed in triplicate, and the error bars represent the standard deviation. The significance was determined using Dunnett’s test.

## 4. Conclusions

A new flavonoid glycoside, herbacetin 7-*O*-neohesperidoside (**1**), was isolated from Ephedra herb extract, an important crude drug in Kampo products, and its structure was elucidated on the basis of spectroscopic and chemical evidence. Nine known compounds were also obtained from the extract and characterized as *trans*-cinnamic acid (**2**), syringin (**3**), catechin (**4**), epicatechin (**5**), symplocoside (**6**), pollenitin B (**7**), herbacetin 7-*O*-glucoside (**8**), kaempferol 3-*O*-rhamnoside 7-*O*-glucoside (**9**), and isovitexin 2-*O*-rhamnoside (**10**). Among the known compounds obtained in the present study, compounds **3**, **6**, **7**, **9**, and **10** were isolated for the first time from the Ephedra species. We also determined that herbacetin (**11**), which is an aglycone of **1**, is one of the active components associated with the antitumor expression shown by Ephedra herb extract.
